# Genetic diversity and population structure of local and exotic sheep breeds in Jordan using microsatellites markers

**DOI:** 10.14202/vetworld.2018.778-781

**Published:** 2018-06-09

**Authors:** Khaleel I. Jawasreh, Mustafa M. Ababneh, Zuhair Bani Ismail, Abdel Mon’em Bani Younes, Ibrahem Al Sukhni

**Affiliations:** 1 Department Animal Production, Faculty of Agriculture, Jordan University of Science and Technology, Irbid, P. O. Box 2210, Jordan; 2 Department of Basic Veterinary Medical Sciences, Faculty of Veterinary Medicine, Jordan University of Science and Technology, Irbid, P. O. Box 2210, Jordan; 3 Department of Veterinary Clinical Sciences, Faculty of Veterinary Medicine, Jordan University of Science and Technology, Irbid, P. O. Box 2210, Jordan

**Keywords:** genetic diversity, Jordan, microsatellites, sheep breeds

## Abstract

**Aim:**

This study was conducted to study the genetic and population structure of local (Awassi) and exotic (Romanov, Charollais, Assaf, Awassi, and Suffolk) sheep breeds in Jordan using eight microsatellite markers.

**Materials and Methods:**

A total of 125 sheep were used (25 from each breed) in the study. The number of alleles (A), the mean values of observed (Ho) and expected (He) heterozygosity, polymorphism information content (PIC), fixation index as a measure of heterozygote deficiency or excess, and Hardy–Weinberg equilibrium (HWE) were analyzed using PopGen and CERVUS softwares. Nei’s standard genetic distances among breeds and dendrogram of unweighted pair group method with arithmetic mean (UPGMA) were calculated and constructed using PopGen software.

**Results:**

A total of 40 alleles were detected with an average number of alleles of 5. The mean Ho value was higher than the mean He value for all breeds. Awassi breed showed the highest average PIC value while Romanov had the lowest. There was a significant (p<0.05) deviation from HWE at each locus within and between breeds. Deviations from HWE were found to be highly significant for all markers except OARFCP304 locus. The genetic distance estimates revealed a close relationship between Romanov and Charollais and between Awassi and Charollais. In the UPGMA dendrogram, Charollais, Romanov, and Awassi breeds were placed together in one main cluster while Assaf was in a different subcluster. Awassi was placed alone in a second main cluster.

**Conclusion:**

Results of this study offer insight toward the genetic conservation of the studied breeds and a base on which breeding plans can be made.

## Introduction

Sheep raising is considered an important source of livelihood in Jordan [1]. The local sheep breed Awassi is well adapted to the semi-arid conditions of Jordan and is considered the most common of all sheep breeds. It is known to resist many diseases and to produce modest amount of good quality meat and milk. Genetic improvement of Awassi has been practiced using selection and crossbreeding [2]. Several other imported breeds also exist in Jordan such as Assaf, Suffolk, Romanov, and Charollais. Due to indiscriminate breeding practices, a great level of genetic diversity or lack off is expected among all sheep breeds in Jordan.

Microsatellites are commonly used to estimate the genetic diversity among different farm animal species [3,4]. Several studies had been investigated the genetic diversity, relatedness, gene mapping, and paternity in goats, sheep, and other animals species using microsatellites markers [5-9].

In Jordan, genetic similarity and distance have been reported within and between Awassi sheep lines (Sagri, black face, and Najdi breeds) using random amplified polymorphic DNA markers [10].

The genetic structure, genetic diversity, and relatedness between Awassi sheep and four of the most common imported sheep breeds Assaf, Suffolk, Romanov, and Charollais were never been studied before. Therefore, the aim of this study was to investigate the genetic structure and variability of Romanov, Charollais, Assaf, Awassi, and Suffolk sheep breeds in Jordan.

## Materials and Methods

### Ethical approval

All experimental procedures in this study were approved (Approval No. 16/4/21/70) by the Jordan University of Science and Technology Animal Use and Care Committee.

### Animals

A total of 125 blood samples were collected from five different sheep breeds (25 samples each) including Awassi, Romanov, Charollais, Assaf, and Suffolk. Whole blood was collected by jugular vein puncture using Vacutainer blood collection tubes containing EDTA and stored at 4°C until analysis.

### DNA extraction

DNA was extracted from whole blood samples using the E. Z. N. blood DNA extraction kit according to manufacturer’s recommendations (Omega Bio-tek, Inc., USA). The extracted DNA was stored at −20°C until further analysis.

### Polymerase chain reaction (PCR) procedure

DNA samples were amplified with eight microsatellite markers (Table-1). The PCR reaction was performed for each locus in 20 µl reactions consisted of 10 µl DNase free water, 2 µl of genomic DNA (20 ng), 2 µl of each of forward and reverse primers, and 4 µl (5 U/µl) of master mix (Hot Firepol, Solis Biodyne, Estonia). The PCR program was carried out at 95°C for 5 min, followed by 35 cycles of 95°C for 30-45 s, annealing for 30-90 s, extension at 72°C for 30-60 s, and final extension at 73°C for 5-30 min. After obtaining the targeted fragment of DNA, the PCR products were mixed 1:3 with loading buffer (98% formamide, 0.09% xylene cyanole, and bromophenol blue), the mixture of PCR product and loading buffer was denatured at 95°C for 5 min, chilled on ice rapidly, and loaded in 12% polyacrylamide gels (29:1 acrylamide: bis-acrylamide). Alleles that have sizes <50 bp were visualized using 12% polyacrylamide gel electrophoresis. The gel was screened by UV illuminator. A 50 bp DNA ladder was used to estimate allele size base pairs (bp).

**Table-1:** Sequences of microsatellite marker primers, annealing temperature, and allele size.

Locus name	Sequence 5´- 3´	Annealing Temperature (°C)	Size (bp)
MAF214	F: GGGTGATCTTAGGGAGGTTTTGGAGG R: AATGCAGGAGATCTGAGGCAGGGACG	63	174-282
MAF65	F: AAAGGCCAGAGTATGCAATTAGGAG R: CCACTCCTCCTGAGAATATAACATG	60	123-127
OARAE129	F: AATCCAGTGTGTGAAAGACTAATCCAG R: GTAGATCAAGATATAGAATATTTTTCAACACC	56	133-159
ILSTS5	F: GGAAGCAATGAAATCTATAGCC R: TGTTCTGTGAGTTTGTAAGC	55	174-218
OARCP34	F: GCTGAACAATGTGATATGTTCAGG R: GGGACAATACTGTCTTAGATGCTGC	54	112-130
OARJMP58	F: GAAGTCATTGAGGGGTCGCTAACC R: CTTCATGTTCACAGGACTTTCTCTG	61	145-169
LSTS11	F: GCTTGCTACATGGAAAGTGC R: CTAAAATGCAGAGCCCTACC	55	256-294
OARFCB304	F: CCCTAGGAGCTTTCAATAAAGAATCGG R: CGCTGCTGTCAACTGGGTCAGGG	61	150-188

### Genetic data analysis

The Ho and He, fixation index (Fis) as a measure of heterozygote deficiency or excess, PIC, and Hardy–Weinberg equilibrium (HWE) were analyzed using PopGen [11] and CERVUS software [12]. Nei’s standard genetic distances among breeds and dendrogram of unweighted pair group method with arithmetic mean (UPGMA) were calculated and constructed using PopGen software [11,13].

## Results

The total numbers of alleles (A), Ho, He, PIC, and HWE for each of the eight microsatellites used in this study are presented in Table-2. All eight microsatellite loci were successfully amplified and were found polymorphic. A total of 40 alleles were detected. The average number of alleles per locus was 5. The number of alleles per locus ranged from 3 (ILSTS5 and ILSTS11) to 9 (MAF214). The Ho and He values ranged between 0.68 to 1.00 and 0.55 to 0.86, respectively. PIC values ranged from 0.44 to 0.84. There was a significant (p<0.001) deviation from HWE at each locus within and between breeds. When the HWE test was performed for each individual locus, deviations from the HWE were found to be highly significant for all markers except OARFCP304 locus.

**Table-2:** Number of alleles (A), observed Ho, expected He, PIC, and HWE for each of the eight microsatellites

Locus	A	Ho	He	PIC	HWE
ILSTS5	3	1.00	0.61	0.53	***
ILSTS11	3	1.00	0.64	0.57	***
OARCP34	4	1.00	0.73	0.68	***
OARFCP304	8	0.68	0.86	0.84	NS
OARJMP58	5	0.79	0.77	0.73	***
OARAE129	4	1.00	0.55	0.44	***
MAF214	9	1.00	0.83	0.81	***
MAF65	4	1.00	0.65	0.58	***
Mean	5	0.90	0.70	0.65	

*** p<0.001, NS=p>0.05 (using Bonferroni *P* value correction). Ho=Observed heterozygosity, He=Expected heterozygosity, PIC=Polymorphic information contents, HWE=Hardy–Weinberg equilibrium

The number of alleles (A), Ho, He, Fis, and PIC for eight microsatellites in Romanov, Charollais, and Suffolk, Awassi, and Assaf sheep breeds are presented in Table-3. The total number of alleles was highest in Awassi (28) followed by Assaf (26), Romanov (25), Charollais, and Suffolk (24). The average number of alleles per breed was 3, 3, 3, 3.5, and 3.3 for Romanov, Charollais, Suffolk, Awassi, and Assaf, respectively. The average Ho value was higher than the He value for all breeds. The average Ho value for overall loci in Romanov, Awassi, Charollais, Suffolk, and Assaf breeds was 0.9, 0.9, 0.8, 0.9, and 0.9, respectively. Whereas, the average He for all loci in the five breeds was 0.6, 0.6, 0.6, 0.7, and 0.7 for Awassi, Assaf, Charollais, Suffolk, and Romanov, respectively. The average PIC values in all breeds varied from 0.5 (Romanov) to 0.6 (Awassi). Within loci, OARJMP58 showed the highest PIC value (0.8) in Awassi sheep.

**Table-3:** Number of alleles (A), observed (Ho), and expected He, Fi as a measure of heterozygote deficiency or excess, and polymorphic information contents (PIC) for eight microsatellites in Romanov, Charollais, and Suffolk, Awassi, and Assaf sheep breeds.

Locus	Romanov					Charollais					Suffolk					Awassi					Assaf				

	A	Ho	He	Fis	PIC	A	Ho	He	Fis	PIC	A	Ho	He	Fis	PIC	A	Ho	He	Fis	PIC	A	Ho	He	Fis	PIC
ILSTS5	3	1	0.5	−0.9	0.4	3	1	0.6	−0.6	0.5	3	1	0.6	−0.6	0.5	3	1	0.6	−0.6	0.5	3	1	0.6	−0.8	0.4
ILSTS11	3	1	0.6	−0.6	0.5	3	1	0.6	−0.6	0.5	3	1	0.6	−0.6	0.5	3	1	0.7	−0.5	0.6	3	1	0.6	−0.6	0.5
OARCP34	2	1	0.5	−1	0.4	3	1	0.6	−0.7	0.5	2	1	0.5	−1	0.4	3	1	0.7	−0.5	0.6	3	1	0.6	−0.6	0.5
OARFCP304	5	1	0.7	−0.5	0.6	4	0.6	0.7	0.1	0.7	4	0.1	0.5	0.8	0.4	5	0.9	0.8	−0.2	0.7	3	0.6	0.7	−0.6	0.6
OARJMP58	4	0.8	0.6	−0.4	0.5	4	0.7	0.7	0.0	0.7	3	0.7	0.7	0.0	0.6	5	0.9	0.8	−0.1	0.7	4	0.9	0.7	−0.4	0.6
OARAE129	2	1	0.5	−1	0.4	2	1	0.5	−1	0.4	2	1	0.5	−1	0.4	3	1	0.6	−0.8	0.5	3	1	0.6	−0.6	0.5
MAF214	4	1	0.7	−0.4	0.6	3	1	0.6	−0.6	0.6	3	1	0.6	−0.6	0.5	4	1	0.7	−0.4	0.7	4	1	0.7	−0.4	0.7
MAF65	2	1	0.5	−1	0.4	2	1	0.5	−1	0.4	4	1	0.8	−0.3	0.7	2	1	0.5	−1	0.4	3	1	0.6	−0.7	0.5
Average	3	0.9	0.6		0.5	3	0.9	0.6		0.5	3	0.8	0.6		0.5	3.5	0.9	0.7		0.6	3.3	0.9	0.7		0.6

Ho=Observed heterozygosity, He=Expected heterozygosity, PIC=Polymorphic information contents, Fis=Fixation index

The matrix of Nei’s standard genetic distances among the studied sheep breeds is presented in Table-4. There is a close relationship between Romanov and Charollais (0.79). A close relationship was also found between Awassi and Charollais (0.79). Nei’s genetic distance between Romanov and Charollais breeds was similar to the genetic distance between Awassi and Charollais breeds (0.24). The genetic distance between Assaf and Suffolk breeds was 0.53.

**Table-4:** Matrix of Nei’s standard genetic distances among sheep breeds.

Breed	Romanov	Charollais	Assaf	Awassi	Suffolk
Romanov	1	0.79	0.69	0.78	0.66
Charollais	0.24	1	0.72	0.79	0.67
Assaf	0.37	0.32	1	0.68	0.59
Awassi	0.25	0.24	0.39	1	0.77
Suffolk	0.42	0.41	0.53	0.27	1

Nei’s genetic identity (above diagonal) and genetic distance (below diagonal)

**Figure-1:**
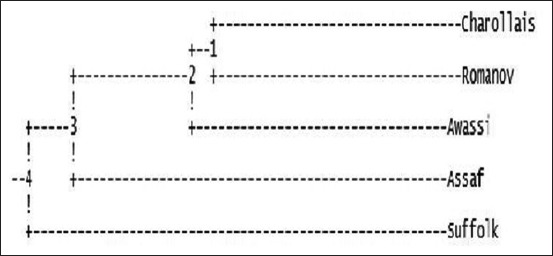
Unweighted pair group method with arithmetic mean dendrogram among Romanov, Charollais, and Suffolk, Awassi, and Assaf sheep breeds.

In the UPGMA dendrogram (Figure-1), two main clusters and two subclusters were identified. In the main clusters, Charollais, Romanov, and Awassi breeds were placed together while Assaf was in the other subcluster. Awassi was placed alone in the second main cluster.

## Discussion

In this study, the genetic contribution of four exotic sheep breeds (Charollais, Romanov, Assaf, and Suffolk) and their relationship with the local Awassi breed was studied for the 1^st^ time using microsatellite markers. The level of variation depicted by the number of alleles at each locus serves as a measure of the impact of each of the studied breeds on differentiation within livestock populations in Jordan. In this study, the number of alleles per locus was ranged from 3 to 9. Similar results were reported in Muzzafarnagri sheep [5] and Bangladesh sheep [7], while a higher number of alleles was detected in Sinai, Rahmani, and Ossimi breeds in Egypt [9] and Kilakarsal sheep [14]. Variation in allele numbers between studies usually indicates differences in studied breeds and markers used. In general, the mean number of alleles detected in each breed or genotype, and the expected heterozygosities are good indicators of the genetic polymorphism within the breed [7].

The mean value of the Ho for all loci in this study was higher than the overall mean value of He. Furthermore, the overall mean value of Ho was higher than the He value for the five studied breeds. These results indicate high genetic variability among studied breeds. Slightly lower Ho values were reported previously in Awassi sheep [15]. It was suggested that higher Ho or He values are highly correlated with higher values of PIC [15].

In this study, Awassi breed showed the highest average PIC value (0.59) while Romanov had the lowest PIC value (0.48). These results are lower than that reported in Kilakarsal sheep breed [14], but similar to those reported in Chokla thin-tailed sheep [16]. Mutation and selection jointly influence the level of polymorphism at microsatellite loci. The high PIC values in this study for the most of microsatellite markers used in this study indicate their suitability in biodiversity evaluation.

All markers in this study revealed a significant departure from HWE except for OARFCP304 marker. This may indicate migration or high mutation rate in microsatellites. It also could be due to artificial selection in the studied breeds [17]. Other factors that may affect HWE values include inbreeding, selection, mutation, and migration. Deviation from HWE at different marker loci has been reported previously in various breeds of sheep [18,19].

Low value of the genetic distance between breeds indicates close relationship or common origin [20]. In this study, Charollais, Romanov, and Awassi breeds showed low genetic distances, while genetic distance was high between Suffolk and the other four studied breeds.

## Conclusion

The genetic diversity and relatedness of local Awassi sheep to four exotic breeds in Jordan were investigated for the 1^st^ time in this study using microsatellite markers. The results indicate high genetic diversity within the studied breeds. At the same time, a close relationship was found between Awassi and Romanov and Awassi and Charollais breeds, whereas distant relationships existed between Awassi and Assaf and between Assaf and Suffolk breeds. This information offers insight toward the genetic conservation of the studied breeds and a base on which breeding plans can be made.

## Authors’ Contributions

KJ: Designed the experiment and performed the laboratory work, the genetic and statistical analysis and manuscript writing. MA: Supervised the molecular experimental work, ZBI: Performed data interpretation and manuscript writing and revision, AMY: Collected samples and performed laboratory work, IAS: Help in the laboratory work. All authors read and approved the final manuscript.
